# Digital food advertising exposure and perceptions among school-age children: a mixed-methods study in Kazakhstan

**DOI:** 10.3389/fpubh.2026.1714870

**Published:** 2026-01-21

**Authors:** Svetlana Rogova, Olga Plotnikova, Marat Kalishev, Nurbek Yerdessov, Aigerim Baimagambetova, Olzhas Zhamantayev

**Affiliations:** 1 School of Public Health, Karaganda Medical University, Karaganda, Kazakhstan; 2 Department of Occupational Hygiene and Occupational Pathology, Omsk State Medical University, Omsk, Russia; 3 School of Public Health and Management, Astana Medical University, Astana, Kazakhstan

**Keywords:** adolescents, children, dietary behaviors, digital, food advertising, Kazakhstan, social media, TikTok

## Abstract

**Background:**

Digital media expose children and adolescents to frequent food advertising, and this content may influence dietary attitudes and choices. This mixed-methods study examined how school-age children in Karaganda, Kazakhstan, perceived food advertising on YouTube, TikTok, and Instagram, and how exposure patterns varied by age.

**Methods:**

The study used a three-day diary to record each food advertisement seen on YouTube, TikTok, and Instagram. Diary data were treated as quantitative records and analyzed with ANOVA, Kruskal-Wallis, chi-square tests, and logistic regression. On day one, children completed a semi-structured interview about their viewing habits and reactions to ads. These interviews were analyzed with thematic analysis.

**Results:**

Diary analysis showed that early adolescents (11–14 years) encountered the most food ads with 14.4 ads/day on average, followed closely by older teens (15–17 years, 13.8 ads/day), whereas younger children (7–10 years) saw far fewer (6 ads/day). TikTok was the dominant source for food ads exposure with Instagram second and YouTube the least. By product, sweetened beverages and fast foods were the most frequently advertised categories. Exposure did not differ overall between morning and evening sessions (*p* = 0.06). Diary entries showed that older adolescents aged 15–17 years reported greater purchase autonomy compared to younger children. Thematic analysis of interviews identified that younger children found advertisements entertaining, 11–14-year-olds reported mixed responses including irritation, and older adolescents displayed critical awareness, often dismissing advertisements as repetitive.

**Conclusion:**

Social media food advertising is pervasive among Kazakhstani children, especially early adolescents. TikTok and Instagram feed flows dominated young viewers’ exposure, with high-calorie products (sugary drinks, fast foods, energy drinks) highly featured. Younger children tended to enjoy ads, whereas older teens generally discounted them, yet older teens’ higher autonomy and consistent exposure to enticing ads suggest potential influence on their choices. Strengthening media literacy and critical thinking among school-aged children should become an important direction in public health to reduce the influence of digital marketing on food-related behavioral decisions.

## 1 Introduction

In many countries, including Kazakhstan, school-age children are increasingly engaged in digital environments, which raises concerns about how digital media content influences their food preferences and behaviors (1, 2). Mobile internet penetration in Kazakhstan reached 148 per 100 people in 2024, indicating widespread digital inclusion and multiple-device use per person (3). National data from 2023 show that 71.8% of children aged 6–10 and 91% of those aged 6–15 used the internet, highlighting strong early digital engagement (4). Globally, youth are similarly connected, according to Pew Research Center about 70% of US adolescents visit YouTube daily and 57% visit TikTok (5), and a WHO survey in Europe, Canada and central Asia found that over one-third of adolescents are in constant online contact with peers, and most have smartphone access (6).

With such high and early exposure, social media platforms have become a key space for commercial food marketing, where companies place targeted advertisements into children’s digital feeds. Nowadays, digital marketing on social media acts as a commercial determinant of health, which refers to company practices and strategies that shape children’s food behavior through the promotion of food products (7, 8). Current studies show that food advertising in digital media is one of the factors that may influence children’s and adolescents’ eating habits and preferences (9, 10). Food and beverage companies are increasingly using social media to advertise, recognizing the platforms’ extensive reach and influence among young people (11). Social media platforms, particularly TikTok and Instagram, are dominant channels for teenagers and play a role in shaping adolescent dietary choices (12, 13).

School-age children often lack strong knowledge about healthy eating, and this gap increases their sensitivity to advertising for unhealthy products (14). Such exposure may shape poor food attitudes and support patterns that continue across adolescence (15, 16). An experimental study in Australia showed that children exposed to unhealthy food advertisements consumed more snacks immediately after viewing them, and combined television plus online game exposure led to an extra 200 kJ at the next snack without later compensation (17). A study from the Netherlands reported that frequent vlog watching was linked to higher intake of unhealthy drinks over 2 years, which reflects patterns seen in traditional marketing (18).

While some parts of the food industry have reduced sugar in selected products by about 20 % and lowered sodium in processed foods by roughly seven to 15 % during the last decade in response to public health targets (19), marketing still places strong focus on items high in fats, sugars, and sodium. These products continue to carry clear links to obesity and cardiovascular risk (20–22). Reports from the United States Food and Drug Administration note that voluntary sodium goals have lowered sodium levels in several categories, yet these levels remain above recommendations, and digital advertising continues to highlight less healthy options (23). A study from Poland showed that children aged 11–13 years often have inadequate fluid intake, partly linked to repeated exposure to sweetened beverage advertising, which may shape poor hydration habits and long-term diet-related problems (24). Empirical evidence shows that aggressive digital marketing of sugary drinks and fast foods is a driver of rising obesity among children (25). A recent study in Kazakhstan found that one in five children aged 6–9 years is overweight, and one in 15 has obesity (26).

Today’s food advertising aimed at young audiences often uses visuals, sounds, popular bloggers, cartoon characters, game-like features, and images of children. These tools draw attention, help viewers remember messages, build trust, and increase the impact of the material while lowering careful reflection (27–29). Advertising appeals can also create emotional ties to brands and give a false sense of thoughtful choice, which may support long-term habits that lack clear reasoning (30). Research shows that adolescents up to 14 years display more openness to advertising than adults and often respond strongly to familiar brands or promotions (31, 32). This pattern becomes even more concerning when considering younger children. Their limited cognitive maturity and early stage of critical thinking make them highly sensitive to manipulative techniques such as bright effects, influencer cues, game-like elements, and hidden promotions (33, 34). Children aged 3 to 5 years can identify packaging and logos and connect them to emotions, even when they do not understand the persuasive aim of advertising (35, 36). By ages 5 to 8, they start to realize that ads promote products, yet they still struggle to judge intent and remain prone to suggestion due to immature reasoning skills (37, 38).

Much of the research on digital food marketing has been conducted in high-income countries. These studies document that popular youth platforms (TikTok, Instagram, YouTube) frequently carry ads for sweets, snacks, sugary beverages, and fast foods, and that teenagers report encountering many such ads each day. By contrast, little is known about the digital food environment for children in Central Asia. Kazakhstan is rapidly digitalizing, but data on how Kazakhstani children encounter and respond to online food advertising are scarce. This study addresses that gap. We examined children’s exposure through their diary entries and analyzed their interview responses to understand how they described and interpreted the food advertisements seen on social media. We focused on age differences, hypothesizing that older adolescents (15–17) would have higher exposure and more critical awareness, whereas younger children (7–10) would react more positively and emotionally. Our objectives were to (1) quantify children’s exposure to different food ads by platform, time of day, and age group, and (2) explore how children of different ages perceive and interpret these digital advertisements.

## 2 Materials and methods

### 2.1 Study design and participant selection

A prospective mixed-methods study examined the content of digital food advertising among school-age children, integrating qualitative and quantitative approaches. Data collection involved semi-structured interviews and three-day observation diaries. The study was conducted from June to July 2025.

The study included 60 school-age children (7–17 years old), residing in the city of Karaganda and studying in general education schools. Four schools were chosen purposively to represent urban public institutions with varied socioeconomic backgrounds. Participants were stratified into three age groups: Group 1 (15–17 years, *n* = 20), Group 2 (11–14 years, *n* = 20), and Group 3 (7–10 years, *n* = 20) on a purposive sampling basis. The 4-year ranges for younger groups (7–10 and 11–14 years) reflect Kazakhstan’s school divisions: Primary Education (grades 1–4, ages 7–10) and Basic Secondary Education (grades 5–9, ages 11–14), while the 3-year range for General Secondary Education (grades 10–11/12, ages 15–17) reflects uniform media engagement (39). Each group was formed with an even distribution by gender (10 boys and 10 girls). The sample size of 60 was determined to balance qualitative depth and quantitative robustness, ensuring sufficient data for thematic analysis and statistical comparisons across age groups (20 per group).

We first conducted an eligibility survey to ensure participants regularly used at least one of the target platforms (YouTube, TikTok, Instagram) with a personal account and spent approximately 15–20 min per session on the app. Children with cognitive or emotional impairments that would interfere with completing the diaries or interviews were excluded. After screening 83 children, 60 meeting criteria were enrolled (the remaining 23 were excluded for low platform usage, lack of device access, or refusal).

### 2.2 Data collection procedure

Observation diaries: On three consecutive days, each child completed an advertisement diary during two fixed 20-min sessions per day in the morning (07:00–10:00) and evening (17:00–21:00). The study was conducted in June and July 2025, during the summer school holidays, so the morning sessions did not overlap with school schedules. During each session, children freely scrolled through content on YouTube, TikTok, and Instagram (approximately 6–7 min per platform per session) using their own devices and accounts. Each participant completed six viewing sessions over the entire observation period (3 days × 2 times), which corresponded to 18 platform episodes. They were instructed to view content naturally, without any specific navigation guidance, to mimic real-world use.

After each session, children recorded every food advertisement they had seen. Food ads were defined broadly (branded videos/posts, influencer endorsements, or any marketing content for a food/beverage). For each ad, children noted the date, time, platform (YouTube/TikTok/Instagram), and food category (e.g., sweetened beverage, fast food, sweets, snacks, dairy, etc.). Also, Groups 1 and 2 also completed quantitative ratings regarding an emotional response (on a 5-point Likert scale from “strongly dislike” to “strongly like”), a “want to try/buy” indicator (yes/no), and who would decide on trying/buying (“self” vs. “parents”). Children also marked how often they usually see each ad (never/sometimes/often) and how often they consume the product (never/sometimes/often). They could add brief comments about their thoughts or feelings on the ad. Parents confirmed the schedule and researcher staff were available by phone to answer any questions. The diary form was piloted with five children in June 2025 to ensure it was age-appropriate.

Interviews: On the first morning, each child participated in a semi-structured interview (15–20 min) in a quiet setting (school or home). Interviews (in Kazakh or Russian) covered typical social media habits (platforms used, viewing frequency/duration), favorite content, knowledge of food brands, attitudes toward different food categories, and reactions to specific ads seen. Open-ended prompts (e.g., “How do you feel when you see a food ad on TikTok?”) were used to describe detailed perceptions. Interview guides were tailored by age group to match language and cognitive level. All interviews were audio-recorded (with permission) and transcribed verbatim. (See Supplementary material S1 for interview questions).

### 2.3 Data analysis

The diary dataset created structured quantitative variables. Children recorded exposure counts, emotional ratings, desire to try or buy, and who would make the purchasing decision. These variables were analyzed with ANOVA, Kruskal-Wallis tests, chi-square tests. We analyzed predictors of self-decision making (self vs. parents) among Groups 1 and 2 (n = 40), including age group and sex as predictors, with logistic regression. All tests were two-tailed, with statistical significance set at *p* < 0.05. We treated diary entries as quantitative records and did not apply qualitative coding to these observations.

The interview transcripts formed the qualitative dataset. These transcripts contained open statements and descriptive accounts of children’s reactions to food advertisements. Two researchers reviewed the transcripts, marked meaning units, and created initial codes (40). They then grouped codes into broader themes. This process followed a stepwise thematic analysis to capture how children interpreted advertisement messages at different ages. To ensure consistency, the data was double-coded and discrepancies were resolved through discussion.

### 2.4 Ethical considerations and quality assurance

The study was approved by the Institutional Review Board of Karaganda Medical University (protocol code 2025-06-17/12, June 17, 2025). Prior to the study, written informed consent was obtained from parents or legal guardians, and verbal consent was obtained from the children. Parents and children were provided with detailed information about the study’s objectives, methods, and conditions, including the need for audio recording of interviews, maintaining observation diaries. Guarantees of anonymity and confidentiality of all collected data were ensured. The study involved no health or behavioral interventions, so direct medical or physiological risks were absent. Minimal socio-psychological risks, such as brief emotional discomfort from viewing advertisements or minor demands on attention and time, were considered. These risks were mitigated through pre-study briefings for parents and children, explaining study aims, methods, and procedures. Participation was voluntary, with the right to withdraw at any time without consequences. Researchers maintained contact, allowing children and parents to report discomfort or seek clarifications. Participants showing psycho-emotional reactions hindering task completion were withdrawn.

To ensure high-quality data collection, we implemented multiple control and standardization measures. Standardized interview guides were used to maintain consistency in data collection procedures across all participants. All researchers underwent specialized training in conducting interviews with children of different age groups to ensure appropriate communication techniques and minimize bias. All qualitative data underwent double-coding by independent researchers, followed by inter-coder reliability assessment to strengthen the validity of analytical results.

## 3 Results

### 3.1 Quantitative results from diaries

Over the three-day observation period, older children encountered substantially more food advertisements than younger ones (Table 1). On average, participants aged 11–14 years viewed 43.20 ads (SD = 6.04), followed by those aged 15–17 years with 41.30 ads (SD = 13.08). Children aged 7–10 years viewed the fewest ads, with a mean of 18.40 (SD = 6.02). Daily averages were 14.40 (SD = 2.01) for the 11–14 years group, 13.77 (SD = 4.36) for 15–17 years, and 6.13 (SD = 2.01) for 7–10 years. A one-way ANOVA confirmed significant differences in total exposure across age groups (*F*(2,57) = 46.91, *p* < 0.001, η^2^ = 0.62). Tukey HSD tests indicated that both adolescent groups had significantly higher exposure than the youngest group (*p* < 0.001), with no significant difference between the 11–14 and 15–17 years groups (*p* = 0.784).

**Table 1:** Mean advertisement exposure by age group over 3 days.

Age group	Mean age (SD)	Total ads (Mean, SD)	Daily ads (Mean, SD)	Per-hour ads (Mean, SD)	Morning ads (Mean, SD)	Evening ads (Mean, sd)
7–10 years	8.20 (1.20)	18.40 (6.02)	6.13 (2.01)	9.20 (3.01)	10.50 (4.53)	7.90 (4.49)
11–14 years	12.60 (1.39)	43.20 (6.04)	14.40 (2.01)	21.60 (3.02)	20.65 (5.37)	22.55 (5.44)
15–17 years	16.45 (0.83)	41.30 (13.08)	13.77 (4.36)	20.65 (6.54)	22.20 (8.43)	19.10 (7.97)

Total ads reflect three-day exposure across all platforms. Per-hour ads are calculated from total ads divided by 2 h of viewing time. Daily advertisement exposure is the mean number of advertisements viewed per day. Morning and evening exposures reflect means for 07:00–10:00 and 17:00–21:00 sessions, respectively. SD, standard deviation.

**Platforms.** TikTok was the leading platform for advertisement exposure across all age groups. Among 11–14 year-olds, 54.05% of all ads were from TikTok, followed closely by 53.89% for 15–17 year-olds. For 7–10 year-olds, TikTok accounted for 37.23% of ads. Instagram ranked second: 35.76% for 11–14 years, 37.84% for 15–17 years, and 38.59% for 7–10 years. YouTube contributed the smallest share: 10.19% for 11–14 years, 8.26% for 15–17 years, and 24.18% for 7–10 years. Kruskal-Wallis tests confirmed significant age group differences in TikTok (*p* < 0.001) and Instagram exposure (*p* = 0.01; Table 2).

**Table 2:** Platform-specific advertisement exposure by age group over 3 days.

Platform	Age group	Morning ads mean (SD)	Evening ads mean (SD)	Proportion of total ads (%)
TikTok	15–17	11.95 (3.09)	10.55 (4.10)	53.89
	11–14	11.40 (2.09)	11.95 (2.33)	54.05
	7–10	4.20 (3.17)	2.65 (2.08)	37.23
Instagram	15–17	9.15 (5.58)	6.65 (1.98)	37.84
	11–14	7.60 (1.76)	7.85 (1.35)	35.76
	7–10	7.15 (2.76)	4.25 (2.45)	38.59
YouTube	15–17	1.55 (1.05)	2.00 (1.26)	8.26
	11–14	2.65 (1.23)	2.75 (1.45)	10.19
	7–10	2.25 (1.59)	1.80 (1.36)	24.18

Morning and evening exposures reflect mean number of advertisements viewed per platform during 07:00–10:00 and 17:00–21:00 sessions, respectively. Proportion of total ads is the percentage of all advertisements (across platforms) attributed to each platform. SD, standard deviation; CI, confidence interval.

**Time of day.** Overall, there was no statistically significant difference in advertisement exposure between morning (07:00–10:00) and evening (17:00–21:00) sessions (Wilcoxon test: Z = −1.884, *p* = 0.060, *r* = 0.24; Table 3). However, age-specific trends emerged. The 11–14 years group had slightly higher exposure in the evening (22.55 ads, SD = 5.44) than in the morning (20.65 ads, SD = 5.37). In contrast, the 15–17 years group reported more ads in the morning (22.20, SD = 8.43) than in the evening (19.10, SD = 7.97). The 7–10 years group maintained consistently low exposure, with 10.50 ads (SD = 4.53) in the morning and 7.90 ads (SD = 4.49) in the evening. Kruskal-Wallis tests revealed significant age group differences in both morning (χ^2^(2) = 30.95, *p* < 0.001, ε^2^ = 0.52) and evening sessions (χ^2^(2) = 40.38, *p* < 0.001, ε^2^ = 0.67).

**Table 3:** Wilcoxon signed ranks test comparing morning and evening advertisement exposure.

Comparison group	N	Mean rank	Sum of ranks	Test statistic	*p*-value
Evening < Morning Ads	32	27.98	895.50	Z = −1.884	0.060
Evening > Morning Ads	20	24.13	482.50		
Ties (Evening = Morning)	8	—	—		

**Food categories.** Sweetened beverages were the most frequently encountered product type across all age groups (Table 4). Participants aged 15–17 years recorded a mean of 13.80 exposures (SD = 8.26), those aged 11–14 years logged 11.75 (SD = 4.01), while 7–10 year-olds reported only 3.60 (SD = 1.98). Fast food was the second most common category, with exposure rates of 8.30 (SD = 4.95) for 15–17 years, 8.40 (SD = 3.25) for 11–14 years, and 4.20 (SD = 2.04) for 7–10 years. Energy drinks were prevalent in older groups (15–17 years: 6.50, SD = 1.85; 11–14 years: 6.35, SD = 2.32) but nearly absent among the youngest (0.30, SD = 0.47). Healthier products appeared infrequently across all age groups, with the 7–10 years group logging only 0.10 (SD = 0.31) exposures. Kruskal-Wallis tests showed significant age differences in fast food (χ^2^(2) = 15.07, *p* = 0.001, ε^2^ = 0.25), energy drink (χ^2^(2) = 40.23, *p* < 0.001, ε^2^ = 0.67), and healthy product exposure (χ^2^(2) = 8.95, *p* = 0.011, ε^2^ = 0.15).

**Table 4:** Food product advertisement exposure and child rating by age group.

Variable	Age group	Mean (SD)	95% CI (lower; upper)
Fast Food	15–17	8.30 (4.95)	5.98; 10.62
	11–14	8.40 (3.25)	6.88; 9.92
	7–10	4.20 (2.04)	3.24; 5.16
Sweetened beverages	15–17	13.80 (8.26)	9.94; 17.66
	11–14	11.75 (4.01)	9.87; 13.63
	7–10	3.60 (1.98)	2.67; 4.53
Snacks (chips, popcorn, crackers)	15–17	1.05 (1.15)	0.51; 1.59
	11–14	1.60 (1.19)	1.04; 2.16
	7–10	2.15 (0.99)	1.69; 2.61
Milk products (frozen yogurt, ice cream)	15–17	1.95 (1.64)	1.18; 2.72
	11–14	2.40 (1.67)	1.62; 3.18
	7–10	1.30 (1.03)	0.82; 1.78
Energy drinks	15–17	6.50 (1.85)	5.63; 7.37
	11–14	6.35 (2.32)	5.26; 7.44
	7–10	0.30 (0.47)	0.08; 0.52
Sweets (chocolate, cookies, pastries)	15–17	4.45 (3.28)	2.91; 5.99
	11–14	5.40 (2.76)	4.11; 6.69
	7–10	5.00 (2.45)	3.85; 6.15
Healthier products (grain bars, oatmeal cookies, puree)	15–17	0.80 (1.01)	0.33; 1.27
	11–14	1.00 (1.38)	0.36; 1.64
	7–10	0.10 (0.31)	−0.04; 0.24
Instant noodles	15–17	2.05 (1.96)	1.13; 2.97
	11–14	2.85 (1.90)	1.96; 3.74
	7–10	0.05 (0.22)	−0.05; 0.15
Combo meals	15–17	2.75 (1.29)	2.15; 3.35
	11–14	3.50 (1.47)	2.81; 4.19
	7–10	1.70 (1.56)	0.97; 2.43
Rating by child^1^	15–17	3.05 (0.69)	2.73; 3.37
	11–14	3.05 (0.69)	2.73; 3.37
	7–10	—	—

^1^Rating scale (1–5) was applied only to participants aged 11–17 years, children aged 7–10 years did not complete numerical ratings.

**Desire to buy and autonomy.** Across adolescents, the average rating for advertising attractiveness was 3.05 (SD = 0.69) on a 5-point scale. No significant difference in ratings was found across age groups (χ^2^(1) = 0.04, *p* = 0.851, ε^2^ = 0.001). Children aged 15–17 more frequently expressed a desire to try or purchase advertised products and indicated that they would make the purchasing decision themselves. Chi-square analysis confirmed an association between age group and decision-making autonomy (χ^2^(1) = 5.23, *p* = 0.022, *φ* = 0.36), while no association was found with sex (χ^2^(1) = 0.11, *p* = 0.744, φ = 0.05; Table 5).

**Table 5:** Summary of ANOVA, Kruskal-Wallis, and chi-square test results.

Analysis	Variable	Test statistic	*p*-value	Effect size
ANOVA	Total ads (3-day)	F(2,57) = 46.91	<0.001	η^2^ = 0.62
	Daily ads	F(2,57) = 46.91	<0.001	η^2^ = 0.62
Kruskal-Wallis	Total morning ads	χ^2^(2) = 30.95	<0.001	ε^2^ = 0.52
	Total evening ads	χ^2^(2) = 40.38	<0.001	ε^2^ = 0.67
	Fast food ads	χ^2^(2) = 15.07	0.001	ε^2^ = 0.25
	Energy drink ads	χ^2^(2) = 40.23	<0.001	ε^2^ = 0.67
	Healthier product ads	χ^2^(2) = 8.95	0.011	ε^2^ = 0.15
	Child rating of ads	χ^2^(1) = 0.04	0.851	ε^2^ = 0.001
Chi-square	Decision maker (age)	χ^2^(1) = 5.23	0.022	φ = 0.36
	Decision maker (sex)	χ^2^(1) = 0.11	0.744	φ = 0.05
	Platform distribution	χ^2^(4) = 75.47	<0.001	Cramer’s V = 0.19

Logistic regression further showed that being in the 15–17 years group significantly predicted independent decision-making (OR = 4.92, 95% CI: 1.20–20.12, *p* = 0.027), while sex was not a predictor (OR = 0.78, *p* = 0.726; Table 6).

**Table 6:** Logistic regression analysis of predictors of self-decision making in purchases.

Variable	B	S. E.	Wald	p	OR	95% CI for OR (lower; upper)
Age group (15–17 vs. 11–14 years)	1.59	0.72	4.90	0.027	4.92	1.20; 20.12
Sex (Female vs. Male)	−0.25	0.70	0.12	0.726	0.78	0.20; 3.10
Constant	−1.27	0.65	3.84	0.050	0.28	—

### 3.2 Interview results

**Figure 1:**
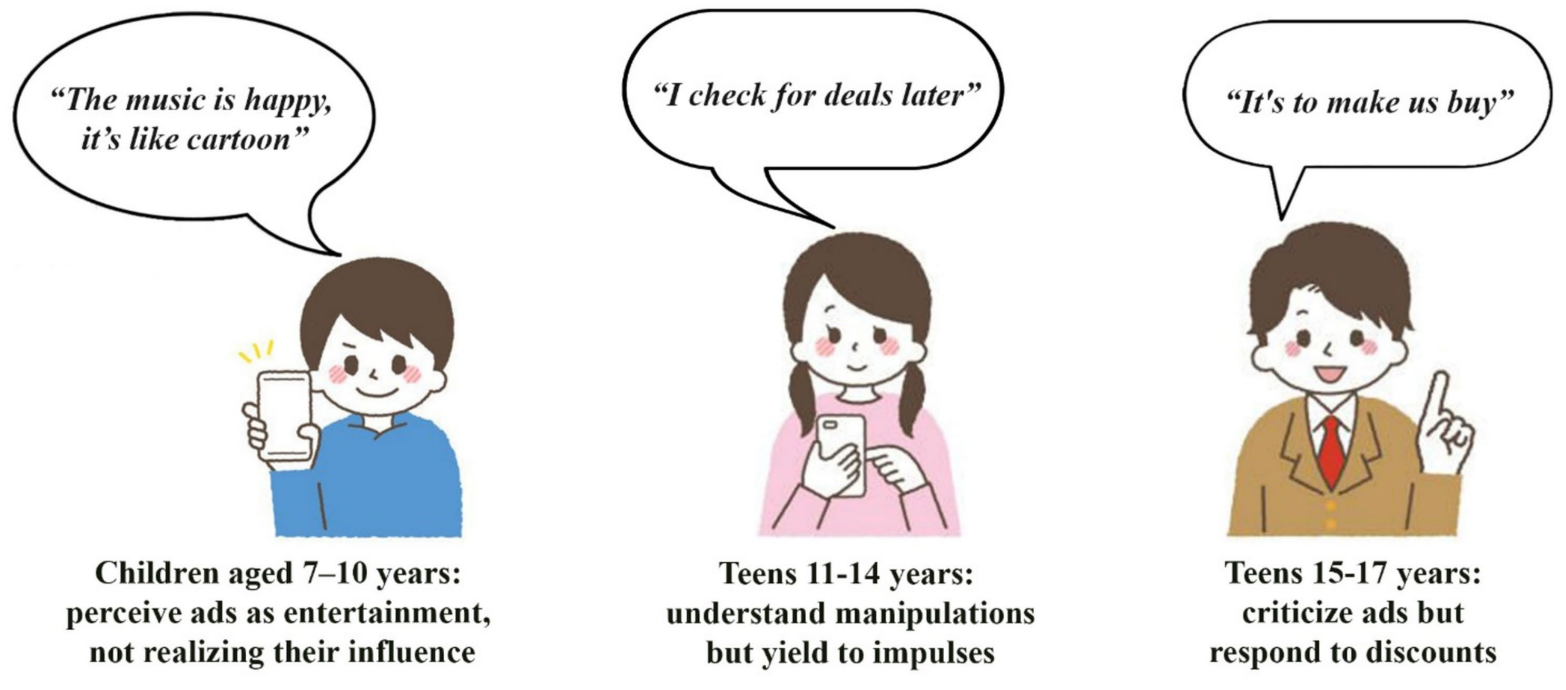
Themes and children’s responses to food advertising on social media by age group.

Semistructured interviews explored how school-age children perceived digital food advertising on YouTube, TikTok, and Instagram. Questions addressed which platforms children used, whether they noticed advertisements during video or gaming sessions, what they liked or disliked, and how they interpreted advertising intent. Thematic analysis identified three key themes aligned with the coding framework: emotional engagement, cognitive awareness of advertising intent, and product interest. These themes reflected age-related patterns and were consistently observed across all three groups (Figure 1).

**Theme 1 – Entertainment/enjoyment (7–10 years)**. Children in the 7–10 years group consistently showed positive emotional responses to food advertisements. All participants in this group described the ads as entertaining, often referring to them as “fun” or resembling cartoons. One 8-year-old boy said, “*The music is fun, like a show*,” and a 9-year-old girl commented that soda commercials were “*just fun to watch*.” Most participants recalled specific jingles or animated visuals, indicating high engagement with the format rather than the persuasive purpose. Although a few children mentioned that “*they want us to buy things*,” understanding of commercial intent was generally absent. Product interest was focused on sweets and brightly packaged beverages. There were no notable differences in reactions between boys and girls.

**Theme 2 – Irritation and mixed reactions (11–14 years).** Among the 11–14 years group, responses were more varied. Emotional engagement was lower than in younger children, and three subthemes were identified: neutral perception, irritation, and selective engagement. Several participants described advertisements as unremarkable or repetitive. A 12-year-old girl said, “*It’s boring, I skip it*,” while a 13-year-old boy noted, “*It interrupts my video, it’s annoying*.” Girls more often expressed fatigue with repetition, whereas boys described the ads as background noise. At the same time, some adolescents reported selective interest in specific promotions or well-known brands. One 14-year-old girl stated, “*I look for deals to buy later*.” Most adolescents demonstrated partial or full awareness of advertising intent. An 11-year-old boy commented, “*They want us to buy stuff*,” and girls often framed product appeal in the context of peer or family influence. Despite the irritation, participants still recalled brand names and product categories, indicating that the content was processed even if not liked.

**Theme 3 – Critical awareness and dismissal (15–17 years).** The 15–17 years group predominantly demonstrated critical understanding and low emotional engagement. Most participants in this group viewed ads as repetitive or irrelevant. A 16-year-old boy remarked, “*I do not care, it’s all the same*,” and a 15-year-old girl said, “*Repeating ads are exhausting*.” Boys frequently noted that the ads lacked substance, while girls found them intrusive. Although emotional responses were generally muted, some adolescents expressed conditional appreciation for ad quality. For example, a 15-year-old girl said, “*Well-made ads look good*.” Product interest was selective. Girls often mentioned promotions or new things “*I look for new flavors or discounts*,” said a 17-year-old girl, while boys were more focused on familiar brands. Importantly, participants in this group showed strong cognitive awareness of advertising strategy and commercial purpose, suggesting they were less susceptible to emotional persuasion than younger participants.

## 4 Discussion

In recent years, digital platforms have transformed food advertising, enabling companies to target children and adolescents with precision (7). Personalized algorithms, behavioral data, and engaging visual formats have allowed food marketing strategies to integrate seamlessly into children’s digital experiences (41). This study examined how school-age children in Kazakhstan are exposed to and interpret food advertising on TikTok, Instagram, and YouTube. The results reveal age-specific exposure patterns, consistent marketing emphasis on unhealthy products, and distinctive psychological responses across developmental stages. These findings have implications for both public health interventions and marketing policy, particularly in emerging digital media environments.

The diary data showed that children aged 11–14 years encountered the most food advertisements, followed closely by those aged 15–17 years. Children aged 7–10 years had substantially lower exposure. These findings confirm that early adolescence (11–14 years) represents a critical period of vulnerability to digital marketing. The peak in exposure coincides with a developmental phase where screen time increases and adult supervision decreases, consistent with previous studies (42–44). Traditional developmental research places the emergence of persuasive intent recognition around age eight (45, 46), but this capacity matures gradually and is context-dependent. Younger children in our study did not consistently recognize persuasive intent, particularly when advertisements were embedded in entertainment content. Their reduced exposure may also reflect greater parental mediation, including supervised use, stricter schedules, or technical restrictions.

We observed a time-of-day shift in exposure patterns with 11–14 year-olds encountered more ads in the evening, when parental oversight tends to lapse, while older adolescents (15–17) had higher morning exposure, possibly due to early-morning device use before school obligations. These trends mirror international research documenting the steady decline in adult regulation and the rise of independent digital behavior across adolescence (47, 48). Notably, the 15–17 year-olds in our study were more likely to report independent purchasing intentions. Logistic regression confirmed that age group significantly predicted decision-making autonomy. This age-related autonomy, paired with high exposure, may compound marketing influence by reducing adult gatekeeping.

TikTok was the most frequent source of food advertisements, especially for adolescents. Among 11–17 year-olds, TikTok accounted for over half of all observed food marketing exposures. This aligns with international data showing that TikTok’s algorithmically curated content stream maximizes ad visibility among youth (49, 50). Instagram followed in frequency, particularly among older teens, while YouTube was less prominent in total exposure.

The prevalence of ads on TikTok and Instagram highlights how marketers have shifted focus toward immersive, visually fast-paced platforms. These platforms allow seamless blending of promotional and organic content, often involving influencers or sponsored challenges. This format is especially effective at reducing ad recognition among younger users (51). Marketing companies may exploit these affordances to deliver branded content that bypasses children’s defenses, particularly when ads feature humor, music, or role models. Our findings reinforce concerns that these platforms serve as high-frequency exposure channels without regulatory buffers.

Across all ages, unhealthy products dominated advertisement exposure. Sweetened beverages were the most frequently logged category, followed by fast food. Older adolescents saw nearly four times as many sugary drink ads as younger children. These figures echo global trends that link youth-targeted marketing to high-sugar beverages, with studies confirming increased consumption and brand loyalty following repeated exposure (37, 52, 53). Fast food ads were nearly as common and showed no significant difference between the two adolescent groups, suggesting a sustained marketing push across ages 11–17.

Energy drinks represented a distinct concern. Although these ads were rare among the 7–10 years group, they appeared frequently for older participants. Both 11–14 and 15–17 year-olds logged multiple energy drink ads, a worrying trend given the established health risks of these products during adolescence (54). Since energy drinks are typically not recommended for minors, the frequency of exposure suggests deliberate targeting or lack of platform regulation. This product category’s digital visibility warrants regulatory attention, especially in Kazakhstan where national advertising standards for online content remain limited.

Perceptions and advertising exposure for sweets were similar across all age groups, possibly indicating a consistent interest in this product category regardless of age. These findings correlate with several international studies that note the dominance of these product groups in marketing targeted at children and adolescents (27, 55).

The inverse trend was observed for healthy products, ads for fruits, dairy items, or whole grains were virtually absent across all age groups. This imbalance may reinforce misconceptions about dietary norms. If children rarely see healthy foods in marketing content, they may perceive them as less desirable or less common. The complete lack of such advertising in the diaries of the 7–10 years group signals missed opportunities for positive reinforcement during early habit formation.

Qualitative data from interviews revealed clear age-dependent differences in how children perceive food advertisements. These perceptions aligned with three distinct psychological profiles derived from our thematic analysis: entertainment, irritation and ambivalence, and critical awareness. For 7–10 year-olds, food ads were viewed as “fun” or “like cartoons.” Children smiled when recalling jingles or animations and rarely questioned the content’s purpose. This entertainment-driven response reflects a low level of persuasion awareness, consistent with studies showing that young children process advertising as narrative or visual play (33). Marketing strategies targeting this group rely heavily on sensory appeal (bright colors, music, animated characters) to build brand familiarity and emotional associations. These techniques create implicit memory cues that persist even without active engagement, making this group highly susceptible to forming early preferences.

Among 11–14 year-olds, reactions were more nuanced. About half of participants expressed irritation or boredom with ads, noting repetition and unwanted interruptions. Others, however, showed selective engagement with ads featuring discounts or familiar brands. This ambivalence reflects developing ad literacy, meaning that these children recognize the persuasive intent but remain partially influenced by product framing. Marketing strategies at this stage often shift toward incentives, such as promotional codes or influencer endorsements, which appeal to emerging consumer agency. These tactics exploit the adolescent desire for peer-relevant consumption without triggering resistance.

Participants aged 15–17 showed strong critical awareness. Most dismissed ads as repetitive or manipulative. Phrases like “It’s all the same” and “I skip them” were common. Still, teens acknowledged that convenience or peer influence could sway their decisions, especially for fast food or sweet drinks. This may highlight that while cognitive defenses may be high, behavioral influence remains possible, particularly through design strategies like autoplay, short-form video, or native influencer content. Marketing companies adapt to this skepticism by embedding product cues in entertaining or user-generated content, formats less likely to be rejected by informed viewers. These psychological profiles reflect strategic adaptation by marketers: from sensory overload in early childhood to social validation in adolescence. The age-specific themes observed in this study map onto known marketing tactics and underscore the need for age-calibrated protections. As UNICEF reports, awareness of advertising does not eliminate its influence, instead, it shifts the mechanisms through which persuasion operates (56).

The dominance of unhealthy products, combined with their integration into high-exposure platforms, supports the classification of food marketing as a commercial determinant of health (19, 32). This system of embedded advertising shapes dietary behavior in ways that extend beyond individual control. We believe that there is a need to treat youth-targeted digital advertising as a structural influence on food choices. Recently, Kazakhstan has initiated positive steps toward regulating school meals and nutritional education (57, 58), but our data suggest that digital marketing remains an unregulated space with considerable influence. National policy could incorporate age-specific marketing limits during peak exposure windows (e.g., early morning and evening hours) and require age-verification filters on food ads. For instance, the UK’s Age-Appropriate Design Code offers a viable model, enforcing data privacy and commercial restraint based on user age (59). Also, from a monitoring perspective, Kazakhstan and other nations would benefit from adopting tools like the WHO CLICK framework to track digital marketing exposure in youth (60). Standardizing metrics for frequency, format, and food category would allow systematic surveillance and policy evaluation.

Educational interventions also have a role. Introducing media literacy modules into primary education can help children critically assess promotional content. Active learning formats such as roleplay, group analysis, app reviews have shown success in reducing susceptibility to food ads (61). These modules could be starting in primary school and progress in complexity by adolescence, reflecting developmental needs.

Our study had several limitations. The diary method relied on children’s self-reports, which may introduce recall bias or result in incomplete entries, and the diaries did not provide qualitative detail beyond the fixed responses. Devices with parental restrictions could limit advertisement exposure, particularly for younger children (7–10 years). Group 1 (15–17 years) spanned only 3 years, potentially reducing developmental variability compared to the 4-year ranges of the other groups. Language barriers may have affected interview responses, as some children required simplified prompts. No compensation was provided, possibly influencing participation motivation (out of 23 potential participants we lost, 18 refused participation). The study focused on YouTube, TikTok, and Instagram, excluding other platforms such as gaming apps. The sample, limited to Karaganda, Kazakhstan, may not be representative of broader populations, however our study could be considered a foundational case for understanding how digital food marketing reaches school-age children in rapidly digitizing, middle-income countries.

## 5 Conclusion

This study explored how school-age children in Karaganda, Kazakhstan, perceive digital food advertising on YouTube, TikTok, and Instagram. Older children faced higher advertisement exposure, particularly on TikTok and Instagram, with sweetened beverages and fast food dominating content. Adolescents showed greater purchase autonomy and cognitive awareness of advertising intent, while younger children viewed ads as entertaining. Girls engaged more with promotions, and boys preferred specific brands. The dominance of unhealthy products in advertising streams and the near absence of healthy alternatives suggest an unbalanced digital food environment that may contribute to poor dietary habits and rising non-communicable disease risks. Public health strategies should include media literacy education beginning in early school years, stricter regulation of youth-targeted food ads on social media, and the integration of marketing exposure into national nutritional surveillance frameworks. These measures will be essential to protect children’s dietary development in increasingly commercial digital spaces.

## Data Availability

The raw data supporting the conclusions of this article will be made available by the authors, without undue reservation.
