# Supplementary material for: Digital food advertising exposure and perceptions among school-age children: a mixed-methods study in Kazakhstan
Source: Front Public Health. 2026 Jan 21;14:1714870. doi: 10.3389/fpubh.2026.1714870 (PMC12868126; doi:10.3389/fpubh.2026.1714870)
Supplement: Supplementary file 1 [file Supplementary_file_1.docx]

Supplementary Material

# Supplementary Data

**Semistructured interview guide**

This interview guide was developed to assess the content and perception of digital food advertising among school-age children (7–17 years) on YouTube, TikTok, and Instagram in Karaganda, Kazakhstan. Questions were tailored to age groups to account for developmental differences, with simpler phrasing for younger children (7–10 years) and more complex prompts for older children (11–17 years). Interviews lasted 15–20 minutes and were conducted in a conversational style to ensure comfort. Questions in italics are optional and may vary based on participants’ responses.

Notes for interviewers:

- Adjust language based on the child’s age and comprehension level.
- Ensure a neutral tone to avoid influencing responses.
- Record responses with permission, or take notes if recording is declined.

Opening questions

1. Which social media platforms do you use (YouTube, TikTok, Instagram)?
2. What kinds of videos or posts do you like to watch on these platforms?
3. How often do you see advertisements for food or drinks when using these platforms?

Perception of food advertising

1. Can you describe a food or drink advertisement you recently saw on social media?
2. What did you think or feel when you watched that advertisement?
3. Do some food advertisements make you want to try the product? *Why or why not?*

Emotional and behavioral responses

1. Do food advertisements ever make you feel happy, excited, or curious? *Can you give an example?*
2. Have you ever asked your parents to buy a food or drink because you saw it in an advertisement?
3. Do you think food advertisements show healthy or unhealthy foods? *How can you tell?*

Age-specific questions

- For younger children (7–10 years)

1. Do you like the colors, music, or characters in food advertisements?
2. Do you talk to your friends about food advertisements you see online?

- For older children (11–17 years)

1. Do you think food advertisements try to convince you to buy things?
2. Can you tell if an advertisement is trying to sell you something unhealthy? *How?*

Closing questions

1. Is there anything else you want to share about food advertisements you see online?
